# S55. MECHANISTIC BASIS OF FRONTO-TEMPORAL TRANSCRANIAL DIRECT CURRENT STIMULATION ON AUDITORY VERBAL HALLUCINATION IN SCHIZOPHRENIA: A MEDIATION ANALYSIS OF COROLLARY DISCHARGE

**DOI:** 10.1093/schbul/sby018.842

**Published:** 2018-04-01

**Authors:** Anushree Bose, Hema Nawani, Sri Mahavir Agarwal, Venkataram Shivakumar, Janardhanan C Narayanaswamy, Devvarta Kumar, Ganesan Venkatasubramanian

**Affiliations:** National Institute of Mental Health and Neurosciences (NIMHANS)

## Abstract

**Background:**

Corollary discharge (CD), ubiquitous throughout the animal kingdom, refers to suppression of sensory consequences arising from self-generated actions. Complex motor acts like covert/overt speech are associated with corollary discharge that helps in ascertaining agency. Auditory verbal hallucinations (AVH) are hypothesized to originate due to failure of corollary discharge in auditory processing system. Transcranial Direct Current Stimulation (tDCS), as an add-on treatment, has been reported to significantly reduce severity of persistent AVH in schizophrenia patients. In this study, we describe mediation analysis findings that strongly support a role for amelioration of corollary discharge deficits as a mechanistic basis for tDCS effects on AVH in schizophrenia.

**Methods:**

27 DSM-IV-TR Schizophrenia patients (SCZ) with persistent AVH despite adequate antipsychotic treatment and 27 healthy controls (HC) underwent neurophysiological assessment for CD. In an event-related potential task, N1 component that reflects cortical responsiveness of auditory cortex to sounds, was elicited and examined in two conditions – i) Talk (with online auricular feedback of self-spoken speech sounds) and ii) Listen (passive playback of recorded self-spoken speech sounds). Corollary discharge index (CDI) was calculated by subtracting Listen condition N1 amplitude from Talk condition N1 amplitude (at FCz). Among these 27 patients, 13 patients participated in a randomized, double-blind, sham-controlled study examining the effect of add-on tDCS on AVH and CDI [5 consecutive days, twice-daily, 20-minute sessions; 2mA; anode: left dorsolateral prefrontal cortex; cathode: left temporo-parietal junction]. Mediation analysis was modelled with tDCS type (Verum vs. Sham) as independent variable, percent change in auditory hallucination rating scale score (AHRS) as dependent variable and percent change in CDI as the mediator. As recommended for small samples, bootstrap estimation approach with 5000 samples was used to examine the indirect effect of independent variable on dependent variable through proposed mediator for significance.

**Results:**

SCZ (Mean±SD: -0.67 ± 1.93) had significantly deficient CDI than HC (1.36 ± 2.18) (t=3.62; p=0.001). Verum tDCS (32.24 ± 16.48) resulted in greater percentage reduction in AHRS than sham (4.79 ± 8.84) (t=3.64, p=0.004). There was a significant increase in CDI (t=2.48; p=0.03) with verum (0.85 ± 1.08) but not sham (-0.55 ± 0.98) tDCS. Percent change in CDI positively correlated with percent change in AHRS from pre-RCT to post-RCT time-point for the entire sample (N=13; ρ=0.55, p=0.05). Regression analysis showed that tDCS type (verum/sham) was a significant predictor of percent change in AHRS (β=-27.46, p=0.003) as well as percent change in CDI (β=-1.40, p=0.033). Percent change in CDI was a significant predictor of percent change in AHRS (β=8.87, p=0.014). When controlled for percent change in CDI, tDCS type ceased to be a significant predictor of percent change in AHRS (β=-15.0, p=0.063). The predictors accounted for approximately 75% of the variance (R2=0.756, p<0.001). Bootstrap estimation results indicate the coefficient of indirect effect to be significant, β=-12.46, SE=6.92, 95% CI=-31.20, -2.79, and significantly different from zero at p<0.05 (two tailed).

**Discussion:**

Fronto-temporal tDCS reduces severity of auditory verbal hallucination in schizophrenia possibly through correction of the deficient corollary discharge. Fronto-temporal network is crucial to self-tagging component of auditory processing and has conspicuous implications for auditory verbal hallucination pathophysiology.

Trial No. CTRI/2014/12/005307 (Clinical Trials Registry–India)

